# Does BMI generated by self-reported height and weight measure up in older adults from middle-income countries? Results from the study on global AGEing and adult health (SAGE)

**DOI:** 10.1186/s40608-015-0074-0

**Published:** 2015-10-26

**Authors:** Theresa E. Gildner, Tyler M. Barrett, Melissa A. Liebert, Paul Kowal, J. Josh Snodgrass

**Affiliations:** Department of Anthropology, University of Oregon, 1321 Kincaid Street, Eugene, OR 97403 USA; World Health Organization, 20 Avenue Appia, CH-1211, Geneva, 27 Switzerland; University of Newcastle Research Centre for Gender, Health and Ageing, HMRI Building Level 4 West Wing, Kookaburra Circuit, New Lambton Heights, NSW 2305 Australia

**Keywords:** Self-report BMI, Measured BMI, Obesity, Older adults, SAGE

## Abstract

**Background:**

Self-reported (SR) body mass index (BMI) values are often used to determine obesity prevalence. However, individuals frequently overestimate their height and underestimate their weight, resulting in artificially lower obesity prevalence rates. These patterns are especially apparent among older adults and overweight individuals. The present cross-sectional study uses nationally representative datasets from five countries to assess the accuracy of SR BMI values in diverse settings.

**Methods:**

Samples of older adults (≥50 years old) and comparative samples of younger adults (18–49 years old) were drawn from five middle-income countries (China, India, Mexico, Russian Federation, and South Africa) in the World Health Organization’s Study on global AGEing and adult health (SAGE). Participant-reported and researcher-obtained height and weight measures were used to calculate SR and measured BMI, respectively. Paired t-tests assessed differences between SR and measured BMI values by country. Linear regressions examined the contribution of measured weight and age to differences between SR and measured BMI.

**Results:**

Significant differences between SR and measured BMI values were observed (*p* < 0.05), but the direction of these discrepancies varied by country, age, and sex. Measured weight significantly contributed to differences between SR and measured BMI in all countries (*p* < 0.01). Age did not contribute significantly to variation in BMI discrepancy, except in China (*p* < 0.001).

**Conclusions:**

These results suggest that SR BMI may not accurately reflect measured BMI in middle-income countries, but the direction of this discrepancy varies by country. This has considerable implications for obesity-related disease estimates reliant on SR data.

## Background

Body mass index (BMI; calculated from individual height and weight values) is a measure commonly used to quantify population-level obesity rates. Self-report (SR) measures of height and weight are often used to calculate individual BMI and demonstrate strong associations with morbidity and mortality [1–3]. The use of SR height and weight values has several advantages, including low cost and ease of collection from a large number of participants. However, despite being associated with disease risk, SR measures may be distorted by participant desire to conform to cultural ideals of beauty and health [4]. BMI based on measurement rather than SR may therefore provide a more accurate BMI value, yet these data are typically more expensive, time-consuming, and intrusive to collect. Consequently, the accuracy of SR measures must be checked to establish the utility of SR BMI as a reliable measure in diverse settings. Confirming the precision of SR measures has particularly important implications in the ongoing struggle to accurately document global increases in obesity. Further, this information is required to design intervention programs that effectively reduce associated disease burden.

Previous studies in wealthy nations demonstrate that individuals often overestimate their height and underestimate their weight, a pattern observed in both sexes [4–10]. For example, Villanueva (2001) found that 25 % of US men and 35 % of US women underreport their weight [11]. This misreporting results in lower obesity prevalence rates when SR data are used to calculate BMI, and these inaccurate values have considerable policy and public health implications. For instance, it is unlikely that SR BMI identifies all overweight and obese individuals, thus impeding the implementation of targeted interventions and the interpretation of lifestyle factors that increase obesity risk [12].

The effect of this misclassification on obesity prevalence data is substantial. A study among Swedish adults demonstrated that obesity prevalence was approximately 5 % lower when SR BMI measures were used in place of measured BMI values [8]. US NHANES III results indicate that BMI based on SR underestimated obesity by 35 % and severe obesity by 31 %; measured BMI was on average 0.6 kg/m^2^ more than SR BMI [13]. Individuals may misreport their BMI due to perceived social pressures to conform to media-enforced cultural standards of desirable height and weight, such as Western ideals of thinness [8].

Individual characteristics, such as measured weight and age, also appear to influence the accuracy of SR BMI values. For example, actual body weight may influence the extent to which an individual underestimates their weight. Hill and Roberts (1998) documented an approximate 0.1 increase in BMI underestimation for every unit increase in measured BMI [7]. Moreover, the overestimation of height and underestimation of weight appears to significantly increase with age, leading to an increased misclassification of overweight and obesity in older adults [14, 15]. This increased likelihood of misreported BMI among older adults appears to be due to decreased stature (a product of vertebral compression), impaired memory, and inability to recognize changes in stature or weight due to poor health [10, 16, 17].

Studies assessing the accuracy of SR BMI have largely been restricted to wealthy countries and younger individuals. The few studies examining these relationships in non-Western nations have produced conflicting results. For instance, weight underestimation and height overestimation (similar to Western populations) have been documented in Brazil, Mexico, Thailand, and China [18–21], while other studies in Mexico and among Brazilian men have observed no significant differences between SR and measured BMI [22, 23]. Further work is therefore required to clarify how differences between SR and measured BMI vary cross-culturally, especially among aging populations. In particular, the relationship between SR and measured BMI should be examined using large, nationally-representative samples to ascertain whether the association between these two measures is the same across different groups, and if not, to determine how this relationship varies in distinctive populations. Information on cross-cultural variation between SR and measured BMI values has the potential to identify key social factors that shape how individual height and weight is perceived and reported in different locations. This information could then be used to elucidate how accurate SR BMI values might be within a specific ecological or cultural setting.

To address these issues, the present study assesses whether SR and measured BMI values differ in older adults across the diverse populations represented in the World Health Organization’s Study on global AGEing and adult health (SAGE) Wave 1 [24]. Data from five middle-income countries (China, India, Mexico, Russian Federation, and South Africa) are used to examine how discrepancies between SR and measured BMI vary across countries. Three hypotheses are tested based on previous research. First, BMI calculated from SR height and weight will be significantly lower than BMI based on measured height and weight at all ages (e.g. older adults will exhibit the same pattern documented previously in younger adults). Second, measured body weight will negatively contribute to the discrepancy between SR and measured BMI, indicating underestimation of SR BMI values in heavier individuals. Third, age will be inversely correlated with SR and measured BMI discrepancy values.

## Methods

### Study design and participants

Nationally-representative samples of older adults (≥50 years old) and comparative samples of adults aged 18–49 years were drawn from China (N = 13,609), India (N = 4,392), Mexico (N = 721), Russia (N = 3,814), and South Africa (N = 1,001) [24]. The complete SAGE Wave 1 dataset also includes participants from Ghana; however, this country was excluded because of a high level of missing self-reported (SR) BMI values. Sampling was based on a stratified, multistage cluster sample design to ensure the full range of living conditions in each country were represented [25]. Face-to-face interviews were used to collect household- and individual-level data. At the time of interviews for SAGE Wave 1, two countries were categorized as lower-middle income (China and India) and three as upper-middle income countries (Mexico, Russia, and South Africa) [26].

### BMI variables

Participants were first asked to report their height and weight during the interview. Many participants failed to provide SR measures (N = 10,395). Still, SR values were obtained from the majority of participants (N = 23,537) and were used to calculate SR BMI as a ratio of weight divided by height squared (kg/m^2^). Trained SAGE interviewers then obtained participant height and weight measurements using standard procedures. Specifically, respondents were asked to wear a single layer of clothing and remove their shoes; participant height was then measured to the nearest 0.1 cm using a stadiometer and weight was recorded to the nearest 0.1 kg using a weighing scale. These values were used to calculate measured BMI (kg/m^2^). Finally, the discrepancy between SR and measured BMI was calculated by subtracting measured BMI from SR BMI; thus, negative discrepancy values correspond to higher measured BMI values relative to SR BMI, while positive discrepancy values reflect higher SR than measured BMI values.

To improve the interpretation of our results, misclassification rates resulting from inaccurate SR BMI values were considered. World Health Organization classifications were used to define BMI categories: underweight (<18.5 kg/m^2^), normal (18.5-24.9 kg/m^2^), overweight (25.0-29.9 kg/m^2^), and obese (≥30 kg/m^2^) [27]. Since the relationships among BMI, body fat percentage, and health risk are different in Asian populations compared to other groups, modified BMI cut-offs for China and India were used: underweight (<18.5 kg/m^2^), normal (18.5-22.9 kg/m^2^), increased risk (23.0-27.5 kg/m^2^), and higher high risk (≥27.5 kg/m^2^) [28].

#### Sociodemographic and health behavior variables

Sex and age were collected as part of the interview. Participants also reported their highest level of education attained, and this variable was dummy coded using “no formal education” as the reference group. Reported annual household income was combined with an index of durable goods ownership, dwelling characteristics, and access to services to create a continuous variable based on long-term wealth status for the household [29]. Total SR physical activity level (PAL) was calculated using interview questions drawn from the Global Physical Activity Questionnaire (GPAQ) [30, 31]. SR time spent in vigorous and moderate exercise for work and leisure were averaged together to create a composite PAL measure (hours/day). Tobacco and alcohol consumption patterns were also determined, sorted into frequency categories, and dummy coded using the “never consumed” categories as reference groups. Finally, following established procedures [8], responses to an overall self-rated general health question (on a 5-point scale from very good to very bad) were dummy coded using the lowest health rating as the reference group.

### Ethical approval

SAGE was approved by the World Health Organization’s Ethical Review Committee. Additionally, partner organizations in each SAGE country obtained ethical clearance through their respective institutional review bodies (the Chinese Center for Disease Control and Prevention Ethical Review Committee, the University of Ghana Medical School Ethics and Protocol Review Committee, the Indian Institutional Review Board for the International Institute for Population Sciences, the Mexican Comisión de Ética en Investigación del Instituto Nacional de Salud Publica, the Russian Academy of Medical Sciences Department of Prophylactic Medicine, and the South African Human Sciences Research Council Ethics Committee). Written informed consent was also obtained from all study participants.

### Statistical analyses

Tests for normality were performed and no violations were observed. Parametric tests were conducted using SPSS version 20; results were regarded as significant at *p* < 0.05. All analyses were run separately by country to better capture inter-population differences.

1. Paired t-tests
  *Hypothesis One – BMI calculated from SR height and weight will be significantly lower than BMI calculated from measured height and weight in both older and younger adults.* Paired t-tests were used to assess differences between SR and measured BMI values. Participants were sorted according to age group and sex, and all analyses were then conducted separately by country. Specifically, differences between SR and measured BMI were evaluated in older men (aged ≥50 years), older women, younger men (aged 18–49 years), and younger women in each country.
2. Linear regressions
  *Hypothesis Two - Measured body weight will negatively contribute to the discrepancy between SR and measured BMI, indicating underestimation of SR BMI in heavier individuals.* Consistent with desired body weight standards seen in wealthy nations, overweight individuals will underreport their weight, resulting in a lower SR BMI value and subsequently a negative discrepancy (SR BMI – measured BMI) due to measured BMI surpassing SR BMI. A linear regression was used to examine if measured weight contributed to variation in the discrepancy between SR and measured BMI among older adults. Sex, age, the education dummy variables, and income (as a continuous variable) were entered in the first step to control for the effects of socioeconomic factors shown to influence accuracy of SR height and weight values [8, 9, 11]. Total PAL and the smoking and drinking dummy codes were entered in the second step of the regression to account for the influence of these variables on the discrepancy between SR and measured BMI [8, 11, 17]. The SR health dummy codes were then entered in the third step of the regression to control for the influence of perceived health on participant discernment of their current height and weight [8, 11]. Measured body weight was entered in the final step. All regressions were conducted separately by country.
  *Hypothesis Three - age will be inversely correlated with discrepancies in SR and measured BMI (i.e. measured BMI will be greater than SR BMI).* A second linear regression assessed if age contributed to discrepancies between SR and measured BMI values. Sex, education dummy codes, and continuous income were entered in the first step of the regression. Total PAL, smoking frequency, and drinking frequency were entered in the second step of the regression. The self-rated health dummy codes were entered in the third step of the regression, and age (as a continuous variable) was entered in the final step of the regression. All regressions were conducted separately by country.

## Results

### Differences between reported and measured BMI by age group

The results of the paired t-tests indicated that significant differences exist between SR and measured BMI values. For example, significant differences between SR and measured BMI in older men (≥50 years old) were observed in India, Russia, and South Africa (*p* < 0.05). Specifically, mean SR BMI was significantly higher than mean measured BMI in India and South Africa, while mean SR BMI was lower than mean measured BMI in Russia (Table 1). Similarly, significant differences between SR and measured BMI in older women were observed in India, Mexico, and Russia (*p* < 0.01). Mean SR BMI in older women was significantly higher than mean measured BMI in India; however, mean SR BMI was lower than mean measured BMI in Mexico and Russia (Table 1).

**Table 1:** Differences between mean self-reported (SR) and measured BMI values (SR BMI – measured BMI). Paired t-tests (2-tailed) assessing differences between self-report (SR) and measured BMI values are presented by sex and country for older (50+ years old) and younger (18–49 years old) individuals, with degrees of freedom (df)

	Older men	Older women	Younger men	Younger women
China	23.5-23.6 = −0.1	24.17-24.22 = −0.6	23.5-23.2 = 0.2	23.1-23.4 = −0.3
	*t*(5623) = −1.03	*t*(6439) = −0.808	*t*(678) = 1.435	*t*(869) = −2.400*
India	23.8-20.7 = 3.1	27.0-21.3 = 5.7	22.7-20.6 = 2.1	24.1-20.7 = 3.4
	*t*(1528) = 8.711***	*t*(964) = 8.815***	*t*(575) = 5.196***	*t*(1325) = 7.534***
Mexico	28.0-28.3 = −0.3	28.8-29.3 = −0.5	28.3-28.7 = −0.4	28.4-29.0 = −0.6
	*t*(286) = −0.837	*t*(308) = −2.740**	*t*(48) = −1.664	*t*(79) = −1.935
Russia	27.0-27.3 = −0.3	29.2-29.7 = −0.4	25.4-26.3 = −0.9	26.0-26.4 = −0.4
	*t*(1235) = −3.540***	*t*(2202) = −6.888***	*t*(143) = −0.914	*t*(234) = −2.252*
South Africa	31.2-29.2 = 2.0	33.4-32.6 = 0.8	30.1-29.1 = 0.9	31.2-29.9 = 1.4
	*t*(406) = 3.016**	*t*(508) = 1.310	*t*(36) = 0.534	*t*(51) = 0.762

Note: Negative values correspond to lower SR BMI than measured BMI values while positive values reflect higher SR than measured BMI values. The number of asterisks indicates the level of significance (* = *p* < 0.05, ** = *p* < 0.01, *** = *p* < 0.001) in chi-square test

Significant differences in SR and measured BMI were also observed among younger men and women (18–49 years old). Among younger men, mean SR BMI was significantly higher than mean measured BMI in India (*p* < 0.001) (Table 1). Among younger women, significant differences were evident in China, India, and Russia (*p* < 0.05). Mean SR BMI was significantly higher than mean measured BMI in India; however, mean SR BMI was significantly lower than mean measured BMI in China and Russia (Table 1).

### Measured body weight and the discrepancy between SR and measured BMI

Measured weight significantly contributed to variation in the discrepancy between reported and measured BMI in China (R^2^ change = 0.043, *p* < 0.001), India (R^2^ change = 0.015, *p* < 0.001), Mexico (R^2^ change = 0.010, *p* = 0.006), Russia (R^2^ change = 0.042, *p* < 0.001), and South Africa (R^2^ change = 0.018, *p* < 0.001), while controlling for key covariates. Specifically, for each unit increase in measured body weight, the discrepancy between SR and measured BMI (SR BMI – measured BMI) became more negative in all of these countries, indicating that SR BMI was lower than measured BMI in heavier individuals (*p* < 0.01) (Table 2).

**Table 2:** Linear regression models for contribution of measured weight to variation in the discrepancy between self-report and measured BMI by country (sexes combined)^a,b^

Variable	Coefficients (SE)	*β*	*p*	Model *r* ^2^ and *p*
BMI Discrepancy (SR BMI – measured BMI)				
China (N = 13,384)				0.043/<0.001***
Constant	5.423 (0.529)		<0.001	
Age	0.009 (0.004)	0.021	=0.028	
Sex	−0.722 (0.126)	−0.071	<0.001	
*Education level*: Completed less than primary	0.241 (0.143)	0.018	=0.091	
Completed primary	0.143 (0.142)	0.011	=0.314	
Completed secondary	0.294 (0.147)	0.024	=0.045	
Completed high school	0.425 (0.165)	0.029	=0.010	
Completed college/university/post-grad	0.356 (0.231)	0.015	=0.124	
Income	0.485 (0.110)	0.042	<0.001	
Total Physical Activity Level	−0.031 (0.014)	−0.020	=0.024	
*Drinking*: Do not currently drink	0.082 (0.217)	0.003	=0.750	
Occasionally	−0.092 (0.148)	−0.006	=0.533	
Moderate/heavy drinker	0.034 (0.139)	0.002	=0.807	
*Smoking*: Do not currently smoke	−0.467 (0.210)	−0.021	=0.026	
Occasionally	−0.431 (0.285)	−0.013	=0.131	
Daily	−0.015 (0.135)	−0.001	=0.911	
*Self-rated health:* Bad	−0.229 (0.332)	−0.017	=0.490	
Moderate	−0.041 (0.324)	−0.004	=0.898	
Good	−0.069 (0.328)	−0.006	=0.832	
Very good	−0.096 (0.383)	−0.004	=0.803	
Measured Weight	−0.095 (0.004)	−0.224	<0.001	
India (N = 4,328)				0.015/<0.001***
Constant	13.074 (2.485)		<0.001	
Age	0.035 (0.018)	0.035	=0.057	
Sex	−0.568 (0.638)	−0.018	=0.373	
*Education Level*: Completed less than primary	−2.225 (0.813)	−0.045	=0.006	
Completed primary	−2.607 (0.746)	−0.060	<0.001	
Completed secondary	−3.188 (0.809)	−0.070	<0.001	
Completed high school	−3.055 (0.890)	−0.064	=0.001	
Completed college/university/post-grad	−3.437 (1.082)	−0.059	=0.002	
Income	−0.113 (0.595)	−0.004	=0.849	
Total Physical Activity Level	−0.036 (0.071)	−0.008	=0.618	
*Drinking*: Do not currently drink	−2.134 (1.151)	−0.029	=0.064	
Occasionally	−0.643 (0.960)	−0.011	=0.503	
Moderate/heavy drinker	−0.935 (1.138)	−0.013	=0.412	
*Smoking*: Do not currently smoke	−0.612 (1.298)	−0.008	=0.637	
Occasionally	−0.548 (1.524)	−0.006	=0.719	
Daily	−0.175 (0.592)	−0.005	=0.768	
*Self-rated health:* Bad	−1.136 (1.889)	−0.026	=0.548	
Moderate	0.895 (1.835)	0.028	=0.626	
Good	0.539 (1.859)	0.016	=0.772	
Very good	0.700 (2.197)	0.009	=0.750	
Measured Weight	−0.173 (0.021)	−0.139	<0.001	
Mexico (N = 718)				0.010/=0.006**
Constant	4.545 (2.680)		=0.090	
Age	−0.021 (0.014)	−0.068	=0.133	
Sex	−0.857 (0.405)	−0.100	=0.035	
*Education Level*: Completed less than primary	−0.711 (0.609)	−0.077	=0.243	
Completed primary	−0.831 (0.641)	−0.085	=0.195	
Completed secondary	−0.077 (0.740)	−0.006	=0.917	
Completed high school	−1.319 (0.988)	−0.063	=0.182	
Completed college/university/post-grad	0.271 (0.737)	0.023	=0.713	
Income	0.452 (0.463)	0.041	=0.329	
Total Physical Activity Level	−0.055 (0.044)	−0.047	=0.218	
*Drinking*: Do not currently drink	0.834 (0.441)	0.082	=0.059	
Occasionally	−0.646 (0.428)	−0.068	=0.132	
Moderate/heavy drinker	−1.386 (0.825)	−0.069	=0.093	
*Smoking*: Do not currently smoke	0.235 (0.421)	0.024	=0.577	
Occasionally	−0.082 (0.654)	−0.005	=0.900	
Daily	−0.023 (0.497)	−0.002	=0.963	
*Self-rated health:* Bad	−0.017 (2.232)	−0.001	=0.994	
Moderate	−0.531 (2.183)	−0.062	=0.808	
Good	−0.212 (2.185)	−0.024	=0.923	
Very good	−0.681 (2.311)	−0.032	=0.768	
Measured Weight	−0.032 (0.012)	−0.111	=0.006	
Russia (N = 3,793)				0.042/<0.001***
Constant	4.223 (0.992)		<0.001	
Age	−0.004 (0.006)	−0.013	=0.524	
Sex	−0.266 (0.156)	−0.035	=0.088	
*Education Level*: Completed less than primary	−1.368 (0.799)	−0.045	=0.087	
Completed primary	−1.301 (0.681)	−0.089	=0.056	
Completed secondary	−1.182 (0.660)	−0.122	=0.073	
Completed high school	−1.310 (0.656)	−0.177	=0.046	
Completed college/university/post-grad	−1.326 (0.667)	−0.145	=0.047	
Income	0.095 (0.167)	0.010	=0.570	
Total Physical Activity Level	0.032 (0.018)	0.029	=0.085	
*Drinking*: Do not currently drink	0.014 (0.194)	0.001	=0.943	
Occasionally	−0.144 (0.153)	−0.019	=0.348	
Moderate/heavy drinker	−0.146 (0.282)	−0.010	=0.604	
*Smoking*: Do not currently smoke	−0.266 (0.208)	−0.023	=0.202	
Occasionally	0.435 (0.449)	0.016	=0.333	
Daily	0.009 (0.197)	0.001	=0.965	
*Self-rated health:* Bad	1.029 (0.509)	0.116	=0.044	
Moderate	1.095 (0.504)	0.145	=0.030	
Good	0.954 (0.529)	0.094	=0.071	
Very good	0.566 (0.826)	0.014	=0.494	
Measured Weight	−0.052 (0.004)	−0.209	<0.001	
South Africa (N = 790)				0.018/<0.001***
Constant	3.822 (5.369)		=0.477	
Age	0.025 (0.046)	0.021	=0.585	
Sex	−0.686 (1.050)	−0.025	=0.514	
*Education Level*: Completed less than primary	2.122 (1.653)	0.068	=0.200	
Completed primary	2.120 (1.722)	0.064	=0.219	
Completed secondary	1.967 (1.897)	0.057	=0.300	
Completed high school	0.468 (2.149)	0.012	=0.828	
Completed college/university/post-grad	2.059 (2.380)	0.047	=0.387	
Income	−1.173 (1.144)	−0.045	=0.306	
Total Physical Activity Level	0.170 (0.159)	0.040	=0.286	
*Drinking*: Do not currently drink	2.116 (7.832)	0.010	=0.787	
Occasionally	0.896 (1.490)	0.024	=0.548	
Moderate/heavy drinker	1.227 (1.757)	0.028	=0.485	
*Smoking*: Do not currently smoke	−0.311 (1.967)	−0.006	=0.874	
Occasionally	−0.356 (2.856)	−0.005	=0.901	
Daily	−2.014 (1.374)	−0.060	=0.143	
*Self-rated health:* Bad	1.244 (3.886)	0.038	=0.749	
Moderate	2.428 (3.802)	0.090	=0.523	
Good	3.014 (3.855)	0.102	=0.435	
Very good	5.418 (4.281)	0.098	=0.206	
Measured Weight	−0.095 (0.025)	−0.143	<0.001	

^a^Comparisons are statistically significant at: * = *p* < 0.05, ** = *p* < 0.01, *** = *p* < 0.001

^b^Reference groups used in the creation of dummy codes for each categorical variable:

(i) Education levels = no formal education

(ii) Drinking levels = never consumed alcohol

(iii) Smoking levels = never used tobacco

(iv) Self-rated health = very bad

### Age and the discrepancy between SR and measured BMI

In all countries except China, age contributed a non-significant amount of variation to the BMI discrepancy (Table 3). In China, for each unit increase in age, the discrepancy between SR and measured BMI increased (B = 0.015, *p* < 0.001), indicating that SR BMI was higher than measured BMI in older adults. However, the added variation explained by the addition of age to the model was very minor (R^2^ change = 0.001), indicating that this very small but statistically significant finding is likely due to the large sample size.

**Table 3:** Linear regression models for contribution of age to variation in the discrepancy between self-report and measured BMI by country (sexes combined)^a,b^

Variable	Coefficients (SE)	*β*	*p*	Model *r* ^2^ and *p*
BMI Discrepancy (SR BMI – measured BMI)				
China (N = 13,384)				0.001/<0.001***
Constant	−0.632 (0.475)		=0.184	
Sex	−0.036 (0.125)	−0.004	=0.771	
*Education Level*: Completed less than primary	0.096 (0.146)	0.007	=0.511	
Completed primary	−0.131 (0.145)	−0.010	=0.368	
Completed secondary	−0.150 (0.149)	−0.012	=0.314	
Completed high school	0.009 (0.168)	0.001	=0.955	
Completed college/university/post-grad	−0.112 (0.235)	−0.005	=0.634	
Income	0.136 (0.111)	0.012	=0.223	
Total Physical Activity Level	−0.023 (0.014)	−0.015	=0.102	
*Drinking*: Do not currently drink	0.101 (0.222)	0.004	=0.650	
Occasionally	−0.068 (0.151)	−0.004	=0.651	
Moderate/heavy drinker	0.131 (0.142)	0.009	=0.355	
*Smoking*: Do not currently smoke	−0.483 (0.215)	−0.022	=0.025	
Occasionally	−0.369 (0.291)	−0.012	=0.205	
Daily	0.173 (0.137)	0.015	=0.209	
*Self-rated health:* Bad	−0.391 (0.339)	−0.029	=0.249	
Moderate	−0.239 (0.331)	−0.023	=0.470	
Good	−0.209 (0.335)	−0.019	=0.533	
Very good	−0.162 (0.391)	−0.007	=0.679	
Age	0.015 (0.004)	0.036	<0.001	
India (N = 4,328)				0.001/0.072
Constant	4.750 (2.286)		=0.038	
Sex	0.673 (0.624)	0.021	=0.281	
*Education Level*: Completed less than primary	−2.193 (0.819)	−0.044	=0.007	
Completed primary	−2.672 (0.752)	−0.062	<0.001	
Completed secondary	−3.449 (0.814)	−0.076	<0.001	
Completed high school	−3.363 (0.896)	−0.070	<0.001	
Completed college/university/post-grad	−4.199 (1.086)	−0.072	<0.001	
Income	−1.269 (0.582)	−0.040	=0.029	
Total Physical Activity Level	−0.053 (0.072)	−0.012	=0.463	
*Drinking*: Do not currently drink	−2.035 (1.160)	−0.028	=0.079	
Occasionally	−0.678 (0.968)	−0.011	=0.483	
Moderate/heavy drinker	−0.983 (1.147)	−0.014	=0.391	
*Smoking*: Do not currently smoke	−0.387 (1.307)	−0.005	=0.767	
Occasionally	−0.446 (1.563)	−0.005	=0.771	
Daily	0.183 (0.595)	0.006	=0.759	
*Self-rated health:* Bad	−0.985 (1.903)	−0.022	=0.605	
Moderate	0.557 (1.849)	0.017	=0.763	
Good	0.181 (1.873)	0.005	=0.923	
Very good	0.029 (2.213)	<0.001	=0.989	
Age	0.033 (0.018)	0.034	=0.072	
Mexico (N = 718)				0.002/=0.264
Constant	1.752 (2.489)		=0.482	
Sex	−0.583 (0.394)	−0.068	=0.139	
*Education Level*: Completed less than primary	−0.841 (0.610)	−0.091	=0.168	
Completed primary	−0.903 (0.643)	−0.092	=0.160	
Completed secondary	−0.065 (0.744)	−0.005	=0.930	
Completed high school	−1.351 (0.992)	−0.065	=0.174	
Completed college/university/post-grad	0.250 (0.740)	0.021	=0.763	
Income	0.253 (0.459)	0.023	=0.582	
Total Physical Activity Level	−0.046 (0.044)	−0.040	=0.306	
*Drinking*: Do not currently drink	0.749 (0.442)	0.074	=0.090	
Occasionally	−0.654 (0.430)	−0.069	=0.129	
Moderate/heavy drinker	−1.257 (0.827)	−0.062	=0.129	
*Smoking*: Do not currently smoke	0.187 (0.422)	0.019	=0.658	
Occasionally	−0.198 (0.655)	−0.012	=0.763	
Daily	0.006 (0.499)	0.001	=0.990	
*Self-rated health:* Bad	0.025 (2.242)	0.002	=0.991	
Moderate	−0.503 (2.193)	−0.058	=0.819	
Good	−0.171 (2.195)	−0.019	=0.938	
Very good	−0.607 (2.321)	−0.028	=0.794	
Age	−0.015 (0.013)	−0.047	=0.264	
Russia (N = 3,793)				<0.001/0.980
Constant	−0.477 (0.942)		=0.612	
Sex	0.006 (0.158)	0.001	=0.972	
*Education Level*: Completed less than primary	−1.166 (0.816)	−0.039	=0.153	
Completed primary	−1.154 (0.696)	−0.079	=0.097	
Completed secondary	−1.049 (0.674)	−0.108	=0.120	
Completed high school	−1.280 (0.670)	−0.173	=0.056	
Completed college/university/post-grad	−1.233 (0.681)	−0.135	=0.070	
Income	−0.064 (0.170)	−0.007	=0.704	
Total Physical Activity Level	0.033 (0.019)	0.031	=0.079	
*Drinking*: Do not currently drink	−0.004 (0.198)	<0.001	=0.985	
Occasionally	−0.146 (0.157)	−0.020	=0.352	
Moderate/heavy drinker	−0.130 (0.288)	−0.009	=0.652	
*Smoking*: Do not currently smoke	−0.264 (0.213)	−0.023	=0.214	
Occasionally	0.556 (0.459)	0.020	=0.225	
Daily	0.214 (0.201)	0.022	=0.286	
*Self-rated health:* Bad	1.169 (0.520)	0.132	=0.025	
Moderate	1.265 (0.515)	0.168	=0.014	
Good	1.309 (0.539)	0.129	=0.015	
Very good	1.231 (0.842)	0.030	=0.144	
Age	<0.001 (0.006)	−0.001	=0.980	
South Africa (N = 790)				0.001/=0.411
Constant	−2.883 (5.114)		=0.573	
Sex	−0.673 (1.059)	−0.025	=0.526	
*Education Level*: Completed less than primary	1.763 (1.665)	0.056	=0.290	
Completed primary	1.687 (1.733)	0.051	=0.331	
Completed secondary	1.502 (1.909)	0.043	=0.432	
Completed high school	0.046 (2.164)	0.001	=0.983	
Completed college/university/post-grad	1.257 (2.391)	0.029	=0.559	
Income	−1.857 (1.139)	−0.071	=0.103	
Total Physical Activity Level	0.116 (0.160)	0.027	=0.467	
*Drinking*: Do not currently drink	0.265 (7.884)	0.001	=0.973	
Occasionally	1.107 (1.502)	0.030	=0.461	
Moderate/heavy drinker	1.876 (1.764)	0.043	=0.288	
*Smoking*: Do not currently smoke	−0.117 (1.983)	−0.002	=0.953	
Occasionally	−0.170 (2.880)	−0.002	=0.953	
Daily	−1.406 (1.377)	−0.042	=0.308	
*Self-rated health:* Bad	0.622 (3.916)	0.019	=0.874	
Moderate	1.618 (3.829)	0.060	=0.673	
Good	2.597 (3.997)	0.088	=0.504	
Very good	5.427 (4.318)	0.098	=0.209	
Age	0.038 (0.04)	0.032	=0.411	

^a^Comparisons are statistically significant at: * = *p* < 0.05, ** = *p* < 0.01, *** = *p* < 0.001

^b^Reference groups used in the creation of dummy codes for each categorical variable:

(i) Education levels = no formal education

(ii) Drinking levels = never consumed alcohol

(iii) Smoking levels = never used tobacco

(iv) Self-rated health = very bad

## Discussion

This study provides a unique examination of the relationship between SR and measured BMI in large samples of older individuals residing in five middle-income countries. The present study found mixed support for the hypotheses. Significant differences between SR and measured BMI values were observed, but the direction of these discrepancies varied by country, age, and sex. Measured body weight contributed to variation in SR and measured BMI differences in all five countries, suggesting that heavier individuals are more likely to underestimate their BMI. Finally, age significantly contributed to variation in BMI discrepancies only in China.

### Differences between SR and measured BMI by country

Older men in India and South Africa reported significantly higher SR BMI than measured BMI values. These findings did not follow the typical pattern observed in wealthier nations (where individuals are more likely to underestimate their BMI); only in Russia did older men significantly underestimate their BMI. Moreover, older women in India overestimated their BMI, whereas only older women in Mexico and Russia underestimated their BMI. These mixed findings were also apparent among younger adults. Younger men significantly overestimated their BMI in India, but in no country did younger men exhibit the expected pattern of underestimating their BMI. Younger women in India also significantly overestimated their BMI; however, younger women in China and Russia underestimated their BMI. Interestingly, at all ages, both men and women systematically overestimated their BMI in India. Similarly, in all age groups (except young men), both men and women significantly underestimated their BMI in Russia.

These findings suggest that cultural differences may influence the accuracy of SR BMI values. For instance, among the five countries examined, the prevalence of underweight by measured BMI is highest in India (Table 4); these individuals are therefore more likely to have a lower BMI than participants living in the other SAGE countries and may estimate BMI values above these relatively low measures. Conversely, more economically developed nations (like Russia) may exhibit diets, activity patterns, and body composition more similar to wealthier nations and consequently underestimate their BMI as has been observed in high-income countries.

**Table 4:** Percentage of population categorized as underweight, normal weight, overweight, or obese based on measured and self-report BMI values. Data are presented by country with number of cases in each category

	China	India	Mexico	Russia	South Africa
Measured BMI: Underweight	4.6 % (640)	34.8 % (3795)	0.9 % (21)	1.0 % (40)	3.9 % (155)
Self-report BMI: Underweight	5.1 % (697)	27.1 % (1210)	1.1 % (8)	1.1 % (47)	2.8 % (29)
Measured BMI: Normal Weight	39.9 % (5569)	41.6 % (4543)	25.9 % (631)	25.1 % (979)	26.0 % (1039)
Self-report BMI: Normal Weight	43.3 % (5949)	39.9 % (1781)	27.7 % (211)	27.1 % (1121)	23.6 % (243)
Measured BMI: Overweight	41.8 % (5845)	16.7 % (1821)	40.7 % (993)	40.9 % (1594)	27.7 % (1106)
Self-report BMI: Overweight	39.3 % (5399)	17.3 % (773)	42.3 % (322)	41.5 % (1720)	27.9 % (287)
Measured BMI: Obese	13.7 % (1920)	6.9 % (756)	32.5 % (793)	32.9 % (1280)	42.5 % (1700)
Self-report BMI: Obese	12.3 % (1688)	15.7 % (699)	28.9 % (220)	30.3 % (1255)	45.7 % (470)

Previous studies have documented multiple sociocultural factors that influence the likelihood of inaccurate SR BMI values. Individual perceptions of body weight in relation to socially-defined desirable weight norms have been linked to both under- and over-reporting BMI, such that overweight individuals tend to underreport their weight, while underweight individuals tend to overestimate their actual weight [32]. Further, previous findings document a positive relationship between difference in participant measured weight and average measurements for their reference group (based on sex and age) and individual likelihood of misreporting weight to conform to these reference values [33].

Thus, socially distinct references of height and weight may have influenced the likelihood of a participant providing imprecise SR BMI values in the present study, and the direction and magnitude of these differences likely varied by population based on culturally unique height and weight norms. Further, these social norms are likely influenced by changes in national level of economic development. For example, increased levels of economic development are significantly associated with changes in social standards of beauty (possibly due to increased Western media exposure), ultimately resulting in individuals becoming more concerned with their weight relative to these new social norms [34, 35]. This could explain why underweight individuals in India would overestimate their BMI, while overweight individuals in other populations underestimate their BMI; in each case, participants were inaccurately reporting values closer to the social norm or the population mean in order to avoid being at the extreme ends of the weight distribution.

These findings have implications for the correct classification of participants into BMI categories and the identification of unhealthy individuals at the extreme ends of the weight spectrum. Interestingly, the degree of BMI category misclassification varied by country. The percentage of obese individuals misclassified in a lower BMI category from SR BMI values ranged from 1.4 % in China to 3.6 % in Mexico (Table 4). Conversely, in India, SR BMI values classified 27.1 % of participants as underweight, while measured BMI classified 34.8 % of these same participants as underweight (Table 4). These findings suggest that the use of SR BMI values may inaccurately measure the prevalence of both over- and under-nutrition in population-level studies, which may have important public health and policy implications. Specifically, inexact measures of global obesity rates preclude the identification of all overweight persons, impacting the implementation of effective weight management programs targeting these individuals. Moreover, imprecise measures of obesity may also result in underestimated healthcare budgets for costs incurred treating obesity-related chronic diseases.

### Measured weight and the discrepancy between SR and measured BMI

Increased measured body weight significantly contributed to the difference between SR and measured BMI as expected (heavier adults underestimated their BMI) in all countries, supporting previous findings in high-income nations. It is worth noting that participant measured height also contributed significantly to variation in the discrepancy between SR and measured BMI in the expected direction; specifically, shorter individuals were more likely to overestimate their height, decreasing their SR BMI value in relation to measured BMI (results not presented). It therefore appears that both participant height and weight contribute to the likelihood of inaccurately reporting these values in SR measures. Still, societal pressure to maintain a culturally-defined preferred weight should not be underestimated. These results suggest that older adults may be compelled to conform to cultural standards of desirable weight, increasing the likelihood of misreporting BMI to comply with these ideals. Although the pressure to adhere to social norms may be greater for younger individuals, previous work suggests that social stigma toward overweight individuals may increase the likelihood of depression in older adults [36]. It is therefore possible that overweight individuals in the present study felt pressured to report weight values closer to culturally-defined body norms due to negative attitudes toward larger body sizes [8, 32, 33].

### Age and the discrepancy between SR and measured BMI

Age did not significantly contribute to variation in this discrepancy, except in China. However, adding age to the regression model for China explained a very small amount of additional variance (R^2^ change = 0.001), indicating that this significant finding is likely due to the large sample size. Overall, these results differ from findings in wealthier nations, which suggest that SR BMI values of older adults are typically more inaccurate than those of younger individuals, largely due to height overestimation as a result of decreased stature, impaired memory, or failure to recognize changes in height or weight because of overall poor health [10, 16, 17]. It is possible that age did not contribute substantially to the regression models in this study because the height and weight of the older participants included in the analyses were consistent over time compared to older adults in wealthier countries, facilitating more accurate SR BMI values at older ages due to minimal long-term changes. Longitudinal data is needed to test these possibilities. Conversely, older adults in SAGE nations may have more accurate knowledge of their height and weight. Future work is needed to explore these factors.

### Limitations

The present study has several limitations. First, these analyses did not control for health conditions known to influence mental acuity (dementia and stroke), which could potentially alter the accuracy of SR weight and height measurements. Second, a large number of SAGE participants did not provide either SR height and weight measurements (N = 10,395 individuals), and many of these individuals also failed to have their height or weight measured by the interviewer. It is possible that obese or underweight individuals with perceived ‘undesirable’ weight may have opted not to respond to these questions, resulting in selective non-response rates that potentially skewed the results. Future analyses will examine the structure of missing data to determine whether the missing values occur at random.

In addition, the SAGE questionnaire did not include detailed information on diet composition; these analyses therefore did not control for individual dietary factors. Self-reported participant ethnicity was also not included in the regression models due to the large number of missing values (N = 2,395). Finally, it was not possible to establish how the precision of individual SR BMI values change over time in this cross-sectional study. Although age generally did not contribute significantly to variation in the discrepancy between SR and measured BMI in the present study, it is possible that advances in age may alter individual accuracy of SR height and weight. SAGE is currently collecting longitudinal data following the progression of these trends over time to address this issue.

## Conclusions

This study documented significant differences in SR and measured BMI that vary by country and often contradict findings from more affluent countries. These results suggest that SR BMI may not accurately reflect measured BMI in middle-income countries, but the direction of this discrepancy varies by country, sex, and age group. This cultural variation in reported BMI has important public health implications and suggests obesity interventions reliant on SR BMI data must carefully assess the validity of SR values based on population. Therefore, how reported BMI values vary in distinct cultures should be considered in future public health interventions and epidemiological studies aimed at decreasing obesity prevalence.

## References

[1] Larsson D, Hemmingsson T, Allebeck P, Lundberg I (2002). Self-rated health and mortality among young men: what is the relation and how may it be explained?. Scand J Public Health.

[2] Heistaro S, Jousilahti P, Lahelma E, Vartiainen E, Puska P (2001). Self rated health and mortality: a long term prospective study in eastern Finland. J Epidemiol Community Health.

[3] Af Sillen U, Nilsson J, Mansson N, Nilsson PM (2005). Self-rated health in relation to age and gender: influence on mortality risk in the Malmo Preventive Project. Scand J Public Health.

[4] Gorber SC, Tremblay M, Moher D, Gorber B (2007). A comparison of direct vs. self‐report measures for assessing height, weight and body mass index: a systematic review. Obes Rev.

[5] Palta M, Prineas RJ, Berman R, Hannan P (1982). Comparison of self-reported and measured height and weight. Am J Epidemiol.

[6] Stewart AW, Jackson RT, Ford MA, Beaglehole R (1987). Underestimation of relative weight by use of self-reported height and weight. Am J Epidemiol.

[7] Hill A, Roberts J (1998). Body mass index: a comparison between SR and measured height and weight. J Public Health.

[8] Nyholm M, Gullberg B, Merlo J, Lundqvist-Persson C, Råstam L, Lindblad U (2007). The validity of obesity based on SR weight and height: Implications for population studies. Obesity.

[9] Danubio ME, Miranda G, Vinciguerra MG, Vecchi E, Rufo F (2008). Comparison of self-reported and measured height and weight: Implications for obesity research among young adults. Econ Hum Biol.

[10] Ramos E, Lopes C, Oliveira A, Barros H (2009). Unawareness of weight and height-the effect on self-reported prevalence of overweight in a population-based study. JNHA.

[11] Villanueva EV (2001). The validity of self-reported weight in US adults: a population based cross-sectional study. BMC Public Health.

[12] Flegal KM, Kit BK, Orpana H, Graubard BI (2013). Association of all-cause mortality with overweight and obesity using standard body mass index categories: a systematic review and meta-analysis. JAMA.

[13] Keith SW, Fontaine KR, Pajewski NM, Mehta T, Allison DB (2011). Use of self-reported height and weight biases the body mass index--mortality association. Int J Obes.

[14] Lawlor DA, Bedford C, Taylor M, Ebrahim S (2002). Agreement between measured and SR weight in older women. Results from the British Women’s Heart and Health Study. Age Ageing.

[15] Dahl AK, Hassing LB, Fransson EI, Pedersen NL (2010). Agreement between SR and measured height, weight and body mass index in old age--a longitudinal study with 20 years of follow-up. Age Ageing.

[16] Kuczmarski MF, Kuczmarski RJ, Najjar M (2001). Effects of age on validity of self-reported height, weight, and body mass index: findings from the Third National Health and Nutrition Examination Survey, 1988–1994. J Am Dietetic Assoc.

[17] Sahyoun NR, Maynard LM, Zhang XL, Serdula MK (2008). Factors associated with errors in self-reported height and weight in older adults. J Nutr Health Aging.

[18] Thomaz PMD, Silva EF, Costa THM (2013). Validade de peso, altura e índice de massa corporal autorreferidos na população adulta de Brasília. Revista Brasileira de Epidemiologia.

[19] Santillan AA, Camargo CA (2003). Body mass index and asthma among Mexican adults: the effect of using SR vs measured weight and height. Int J Obes.

[20] Lim LL, Seubsman SA, Sleigh A (2009). Validity of SR weight, height, and body mass index among university students in Thailand: Implications for population studies of obesity in developing countries. Popul Health Metr.

[21] Zhou X, Dibley MJ, Cheng Y, Ouyang X, Yan H (2010). Validity of SR weight, height and resultant body mass index in Chinese adolescents and factors associated with errors in self-reports. BMC Public Health.

[22] Osuna-Ramírez I, Hernández-Prado B, Campuzano JC, Salmerón J (2006). Indice de masa corporal y percepción de la imagen corporal en una población adulta mexicana: la precisión del autorreporte. Salud Pública de México.

[23] Rech CR, Petrosk EL, Boing O, Babel RJ, Soares MR (2007). Agreement between SR weight and height measurements for the diagnosis of the nutritional status of older residents in Southern Brazil. Rec Bras Med Esporte.

[24] Kowal P, Chatterji S, Naidoo N, Biritwum R, Fan W, Ridaura RL, Maximova T, Arokiasamy P, Phaswana-Mafuya N, Williams S, Snodgrass JJ, Minicuci N, D’Este C, Peltzer K, Boerma JT (2012). Data resource profile: The WHO Study on Global Ageing and Adult Health (SAGE). Int J Epidemiol.

[25] Naidoo N. SAGE Working Paper No. 5 WHO Study on global AGEING and adult health (SAGE) Waves 0 and 1 - Sampling information for China, Ghana, India, Mexico, Russia, and South Africa. World Health Organization 2012. http://www.who.int/healthinfo/sage/SAGEWorkingPaper5_Wave1Sampling.pdf.

[26] The World Bank. World Bank list of economies. http://data.worldbank.org/news/2010-GNI-income-classifications.

[27] WHO [World Health Organization] (2000). Obesity: Preventing and Managing the Global Epidemic.

[28] WHO [World Health Organization] (2004). Appropriate body-mass index for Asian populations and its implications for policy and intervention strategies. J Lancet.

[29] Kowal P, Biritwum R, Haldia KR, Isingo R, Kumogola Y, Mathur A, Naidoo N, Urassa M, Chatterji S. SAGE Working Paper No. 2 WHO Study on global AGEing and adult health (SAGE): Summary results of a pilot in three countries. World Health Organization 2012. http://www.who.int/healthinfo/sage/SAGEWorkingPaper2_Pilot_summary_Oct12.pdf.

[30] Armstrong T, Bull F (2006). Development of the World Health Organization global physical activity questionnaire (GPAQ). J Public Health.

[31] Bull FC, Maslin TS, Armstrong T (2009). Global physical activity questionnaire (GPAQ): nine country reliability and validity study. J Phys Act Health.

[32] Chau N, Chau K, Mayet A, Baumann M, Legleye S, Falissard B (2013). Self-reporting and measurement of body mass index in adolescents: refusals and validity, and the possible role of socioeconomic and health-related factors. BMC Public Health.

[33] Gil J, Mora T (2011). The determinants of misreporting weight and height: the role of social norms. Econ Hum Biol.

[34] Jeffery RW (1996). Socioeconomic status, ethnicity and obesity in women. Ann Epidemiol.

[35] Seubsman SA, Lim LL, Banwell C, Sripaiboonkit N, Kelly M, Bain C, Sleigh AC (2010). Socioeconomic status, sex, and obesity in a large national cohort of 15-87-year-old open university students in Thailand. J Epidemiol.

[36] Palinkas LA, Wingard DL, Barrett-Connor E (1996). Depressive symptoms in overweight and obese older adults: a test of the “jolly fat” hypothesis. J Psychosom Res.

